# Immunohistochemical Expression of Vimentin in Breast Carcinoma and Its Correlation With Histopathological Prognostic Factors

**DOI:** 10.7759/cureus.107767

**Published:** 2026-04-26

**Authors:** Rashmi Jain, Ankit Jain, Saumya

**Affiliations:** 1 Department of Pathology, Government Medical College, Satna, IND; 2 Department of Anatomy, Government Medical College, Satna, IND; 3 Department of Obstetrics and Gynecology, Government Medical College, Satna, IND

**Keywords:** breast carcinoma, histological grade, immunohistochemistry, lymph node status, tumor size, vimentin

## Abstract

Background: Vimentin is a mesenchymal intermediate filament protein and an important marker of epithelial-mesenchymal transition, which has been implicated in tumor invasion, progression, and aggressive biological behavior in breast carcinoma. Its expression in invasive breast carcinoma may correlate with established pathological prognostic factors and thereby provide additional prognostic information.

Objective: To evaluate the expression of vimentin in invasive breast carcinoma of female patients and to determine its correlation with markers associated with adverse histopathological features.

Methodology: This retrospective observational study was carried out in the Department of Pathology, Gandhi Medical College, Bhopal, Madhya Pradesh, India. A total of 70 cases of invasive ductal carcinoma of the breast, not otherwise specified (IDC-NOS), were included. Histopathological evaluation was performed on hematoxylin and eosin-stained sections, and tumor grading was done according to the Modified Bloom-Richardson grading system. Tumor size and lymph node status were categorized according to standard pathological criteria. Immunohistochemical staining for vimentin was performed on representative paraffin-embedded tissue sections. Vimentin expression was assessed as positive or negative based on cytoplasmic staining in invasive tumor cells.

Results: Vimentin expression was observed in 17 of 70 cases (24.28%). An increasing trend of vimentin positivity was seen with increasing tumor size, with positivity in 0 of 3 cases (0.0%) in tumors measuring ≤2 cm, 5 of 28 cases (17.85%) in tumors measuring >2 to ≤5 cm, and 12 of 39 cases (30.76%) in tumors measuring >5 cm. Vimentin positivity also increased with nodal involvement and was seen in 4 of 32 node-negative tumors (12.50%), 3 of 17 N1 tumors (17.64%), 8 of 18 N2 tumors (44.44%), and 2 of 3 N3 tumors (66.66%), although the association was not statistically significant (p = 0.06). A strong association was observed with histological grade: none of the Grade I tumors [0 of 19 cases (0.0%)] were positive, whereas positivity was present in 7 of 38 Grade II tumors (18.42%) and 10 of 13 Grade III tumors (76.92%).

Conclusions: Vimentin expression in invasive breast carcinoma is associated with adverse pathological features, particularly larger tumor size, higher nodal burden, and most strongly, higher histological grade. Its marked expression in poorly differentiated tumors suggests that vimentin may serve as a useful adjunct prognostic marker of aggressive tumor biology in invasive breast carcinoma.

## Introduction

Breast carcinoma remains the most common malignancy among women worldwide. It is a major contributor to cancer-related mortality, particularly in low- and middle-income countries where delayed presentation and biologically aggressive disease are frequent [1,2]. Despite substantial advances in imaging, molecular classification, and systemic therapy, histopathological evaluation continues to play a central role in prognostication and treatment planning in invasive breast carcinoma. Conventional prognostic parameters such as tumor size, axillary lymph node status, and histological grade remain robust and clinically relevant indicators of tumor behavior and patient outcome [3,4].

Breast cancer progression is not determined solely by tumor burden, but also by the intrinsic biological behavior of tumor cells. One of the most important biological events associated with invasion and metastasis is epithelial-mesenchymal transition (EMT), a process in which epithelial tumor cells lose intercellular adhesion and acquire mesenchymal characteristics that facilitate motility, invasiveness, and metastatic spread [5,6]. Among the proteins associated with EMT, vimentin has gained particular importance as a marker of tumor aggressiveness.

Vimentin is a type III intermediate filament protein normally expressed in mesenchymal cells, including fibroblasts, endothelial cells, and leukocytes [7]. In epithelial malignancies, however, aberrant vimentin expression has been increasingly recognized and is thought to reflect dedifferentiation and the acquisition of an invasive phenotype [5,8]. In breast carcinoma, vimentin expression has been associated with increased migratory capacity, altered cytoskeletal organization, tumor progression, and resistance to therapy, making it a potentially useful marker of biologically aggressive disease [6,9].

Several investigators have evaluated the clinicopathological significance of vimentin in breast carcinoma. Early studies demonstrated that vimentin expression is more commonly seen in high-grade invasive ductal carcinomas and in tumors with poor differentiation [10,11]. Subsequent studies reported associations between vimentin positivity and adverse pathological parameters, including higher histological grade, larger tumor size, lymph node metastasis, hormone receptor negativity, and basal-like phenotype [12-15]. However, the published literature is not entirely consistent, and some studies have failed to show a significant association between vimentin expression and tumor size or nodal metastasis [16,17]. These discrepancies may reflect differences in sample size, case selection, tumor subtype composition, antibody methodology, and scoring criteria.

In resource-constrained settings, where access to advanced molecular profiling may be limited, immunohistochemical biomarkers that correlate with established prognostic factors may offer practical value in risk stratification. Assessing vimentin expression in invasive breast carcinoma may therefore provide additional insight into tumor biology beyond routine histomorphology. In particular, its relationship with tumor size, lymph node status, and Modified Bloom-Richardson histological grade may help identify tumors with a more aggressive phenotype.

Hence, the present study was undertaken to evaluate the expression of vimentin in invasive breast carcinoma and to assess its relationship with established histopathological prognostic factors. The study primarily aimed to determine the frequency of vimentin expression in invasive ductal carcinoma of the breast. Additionally, it sought to evaluate the association of vimentin expression with tumor size, lymph node status, and histological grade, and to examine its relationship with adverse histopathological characteristics indicative of aggressive tumor behavior. It was hypothesized that vimentin expression would be more frequently observed in tumors exhibiting adverse histopathological characteristics.

## Materials and methods

Study design and setting

This retrospective observational study (from June 1, 2015, to May 30, 2016) was conducted in the Department of Pathology at Gandhi Medical College, Bhopal, Madhya Pradesh, India. The study was conducted on archived histopathological specimens of female patients diagnosed with invasive breast carcinoma. The work was based on mastectomy specimens received in the department, and the study was undertaken after obtaining approval from the Institutional Ethics Committee (10304.05/MC/7/2015, dated 11/05/2015). The present manuscript represents a focused analysis of the vimentin-related objectives from the thesis dataset and includes only those variables relevant to the current research question.

Study population

A total of 70 cases of invasive ductal carcinoma of the breast, not otherwise specified (IDC-NOS), were included in the study. These cases were selected from mastectomy specimens available in the departmental archives. Only female patients were included, and all selected cases had adequate histopathological material available for review and immunohistochemical evaluation. The study was designed to assess vimentin expression in invasive breast carcinoma and determine its correlation with selected pathological prognostic markers, namely tumor size, lymph node status, and histological grade.

Inclusion and exclusion criteria

Female patients with histopathologically confirmed invasive ductal carcinoma of the breast (NOS) and available formalin-fixed paraffin-embedded tissue blocks were included in the study. Cases were also required to have adequate pathological details regarding tumor size, axillary lymph node status, and histological grade. Male breast carcinoma, histological variants other than invasive ductal carcinoma (NOS), breast carcinoma occurring during pregnancy, and cases previously exposed to chemotherapy or radiotherapy were excluded from the study, as prior treatment could alter both tumor morphology and immunohistochemical staining characteristics.

Data collection

Relevant clinicopathological information was retrieved from the departmental records, pathology archives, and histopathology requisition forms. For the present analysis, only those parameters directly related to the study objectives were considered. These included tumor size, lymph node status, and histological grade. Other variables available in the thesis dataset were not included in this manuscript to maintain focus on the predefined vimentin-related objectives.

Histopathological evaluation

Archived hematoxylin and eosin (H&E)-stained slides and corresponding paraffin blocks of all selected cases were reviewed. Histological confirmation of invasive ductal carcinoma (NOS) was done on routine microscopic examination. Tumor grading was performed according to the Modified Bloom-Richardson grading system, which evaluates the tumor based on tubule formation, nuclear pleomorphism, and mitotic activity and classifies the lesions into Grade I, Grade II, and Grade III [3,18]. Tumor size was categorized according to pathological tumor dimension into T1 (≤2 cm), T2 (>2 to ≤5 cm), and T3 (>5 cm). Similarly, lymph node status was classified as N0, N1, N2, or N3 based on the number of involved axillary lymph nodes, in accordance with standard TNM pathological staging criteria [19]. These pathological parameters were subsequently correlated with vimentin expression.

Immunohistochemistry for vimentin

Immunohistochemical staining for vimentin was performed using a monoclonal mouse anti-human vimentin antibody (Biogenex, San Ramon, CA; clone V9) at a dilution of 1:100, as per the manufacturer’s protocol. Antigen retrieval was carried out using trisodium citrate buffer (pH 6.0) in a pressure cooker. The sections were incubated with the primary antibody for 60 minutes at room temperature, followed by incubation with a secondary detection system using a horseradish peroxidase (HRP)-based polymer detection kit. Visualization was achieved using 3,3′-diaminobenzidine (DAB) as chromogen, and hematoxylin was used as the counterstain.

Known positive control tissues (mesenchymal tissue) and negative controls (sections processed without primary antibody) were included in each staining batch to ensure staining validity.

Vimentin expression was assessed independently by two experienced pathologists who were blinded to clinicopathological data. Only distinct cytoplasmic staining in invasive tumor cells was considered positive. A cutoff value of ≥10% tumor cell staining was used to define positivity, based on previously published studies evaluating vimentin expression in breast carcinoma. Cases showing <10% staining were considered negative. Any discrepancies between observers were resolved by joint review to reach a consensus. Interobserver agreement was assessed qualitatively and showed high concordance between observers.

Composite risk stratification

A composite histopathological risk category was derived based on tumor size, lymph node status, and histological grade to classify cases into low-, intermediate-, and high-risk groups.

Outcome measures

The primary outcome of the study was to determine the frequency of vimentin expression in invasive breast carcinoma. The secondary outcomes included correlations of vimentin expression with tumor size, lymph node status, and Modified Bloom-Richardson histological grade, to evaluate whether vimentin expression was associated with established pathological indicators of aggressive tumor behavior.

Statistical analysis

The collected data were entered into Microsoft Excel and analyzed using SPSS software (version 26, IBM Corp., Armonk, NY). Categorical variables were expressed as frequency and percentage. The association between vimentin expression and clinicopathological prognostic parameters, such as tumor size, lymph node status, and histological grade, was assessed using the chi-square test, with Yates' correction applied where required. The association between categorical variables was assessed using the chi-square test. In cases where expected cell counts were less than 5, Fisher’s exact test was applied as appropriate. A *P*-value <0.05 was considered statistically significant.

## Results

**Figure 1:**
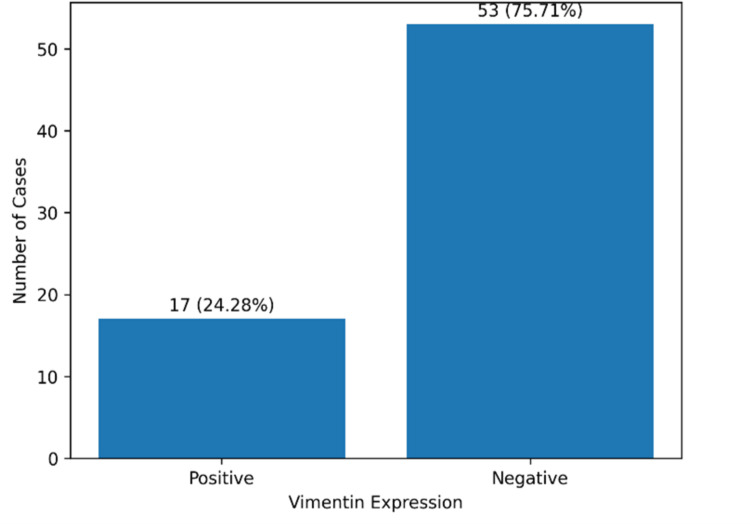
Overall immunohistochemical expression of vimentin in invasive breast carcinoma (n = 70). Bar diagram showing the proportion of vimentin-positive and vimentin-negative cases among the studied invasive breast carcinomas. Vimentin expression was assessed by immunohistochemistry. Values are expressed as number (percentage).

A total of 70 cases of invasive breast carcinoma were evaluated for immunohistochemical expression of vimentin. Overall, 17 cases (24.28%) showed positive vimentin expression, whereas 53 cases (75.71%) were negative, indicating that vimentin positivity was present in nearly one-fourth of the studied tumors (Figure 1). The baseline clinicopathological characteristics of the study population are shown in Table 1.

**Table 1:** Baseline clinicopathological characteristics of the study population (n = 70). Data are presented as number (percentage). Tumor size was categorized according to pathological tumor dimension as T1 (≤2 cm), T2 (>2 cm to ≤5 cm), and T3 (>5 cm). Lymph node status was classified based on the number of involved axillary lymph nodes as N0 (no nodes), N1 (1-3 nodes), N2 (4-9 nodes), and N3 (≥10 nodes), in accordance with standard TNM staging criteria. Histological grading was performed using the modified Bloom-Richardson grading system. n = number of cases; T = tumor size category; N = lymph node status.

Variable	Frequency *n* (%)
Tumor size	
≤2 cm (T1)	3 (4.3)
>2 to ≤5 cm (T2)	28 (40.0)
>5 cm (T3)	39 (55.7)
Lymph node status	
Negative (N0)	32 (45.7)
Positive (N1-N3)	38 (54.3)
Detailed Nodal Status	
N0 (0 nodes)	32 (45.7)
N1 (1-3 nodes)	17 (24.3)
N2 (4-9 nodes)	18 (25.7)
N3 (>10 nodes)	3 (4.3)
Histological grade	
Grade I	19 (27.1)
Grade II	38 (54.3)
Grade III	13 (18.6)

Vimentin expression and tumor size

When correlated with tumor size, vimentin expression demonstrated an increasing trend with increasing primary tumor dimension. None of the tumors in the T1 category (≤2 cm) were vimentin-positive. In contrast, positivity was observed in a proportion of T2 tumors (>2 to ≤5 cm) and became more frequent in T3 tumors (>5 cm), suggesting that vimentin expression tends to be associated with larger tumor size (Table 2).

**Table 2:** Association of vimentin expression with tumor size (n = 70). Pearson’s chi-square test demonstrated a statistically significant association between tumor size and vimentin expression (χ² = 7.1, df = 2, *P* = 0.028).

Tumor size (cm)	Vimentin positive, *n* (%)	Vimentin negative, *n* (%)	Total, *n* (%)	χ²	*P*-value
≤2 (T1)	0 (0.00)	3 (100.00)	3 (100.00)	7.1	0.028
>2 to ≤5 (T2)	5 (17.85)	23 (82.14)	28 (100.00)		
>5 (T3)	12 (30.76)	27 (69.23)	39 (100.00)		
Total	17 (24.28)	53 (75.71)	70 (100.00)		

Vimentin expression and lymph node status

A progressive rise in vimentin positivity was observed with advancing lymph node involvement. Vimentin expression was least frequent in node-negative tumors (N0) and increased across the N1, N2, and N3 categories, indicating a trend toward greater expression with increasing nodal burden (Table 3). When grouped broadly, lymph node-positive tumors demonstrated higher Vimentin positivity than node-negative tumors. However, this association did not achieve statistical significance (Yates χ² = 3.35, df = 1, *P* = 0.06).

**Table 3:** Association of vimentin expression with lymph node status (n = 70). Chi-square test: Yates χ² = 3.35, df = 1, *P* = 0.06.

Lymph node status	Vimentin positive, *n* (%)	Vimentin negative, *n* (%)	Total, *n* (%)
N0 (0 nodes)	4 (12.50)	28 (87.50)	32 (100.00)
N1 (1-3 nodes)	3 (17.64)	14 (82.35)	17 (100.00)
N2 (4-9 nodes)	8 (44.44)	10 (55.55)	18 (100.00)
N3 (>10 nodes)	2 (66.66)	1 (33.33)	3 (100.00)
Total	17 (24.28)	53 (75.71)	70 (100.00)

Vimentin expression and histological grade

Significant association with higher histopathological risk categories was observed between vimentin expression and modified Bloom-Richardson histological grade. None of the Grade I tumors expressed vimentin, while only a minority of Grade II tumors were positive. In contrast, vimentin positivity was seen in the majority of Grade III tumors, indicating that vimentin expression increased markedly with worsening histological differentiation (Table 4). This pattern suggests that vimentin is predominantly associated with poorly differentiated and biologically aggressive carcinomas.

**Table 4:** Association of vimentin expression with modified Bloom-Richardson histological grade (n = 70). Statistical test: Pearson’s Chi-square test; χ² = 27.19, df = 2, *P* < 0.001.

Histological grade	Vimentin positive, *n* (%)	Vimentin negative, *n* (%)	Total, *n* (%)	χ²	*P*-value
Grade I	0 (0.00)	19 (100.00)	19 (100.00)	27.19	<0.001
Grade II	7 (18.42)	31 (81.57)	38 (100.00)		
Grade III	10 (76.92)	3 (23.07)	13 (100.00)		
Total	17 (24.28)	53 (75.71)	70 (100.00)		

Composite risk stratification

A statistically significant association was observed between vimentin expression and composite histopathological risk categories (χ² = 22.84, *P* < 0.001), with the highest positivity seen in the high-risk group (Table 5).

**Table 5:** Association of vimentin expression with composite histopathological risk category (n = 70). Vimentin expression was assessed by immunohistochemistry and categorized as positive or negative based on cytoplasmic staining in invasive tumor cells. Risk categories were derived from established histopathological prognostic factors: low risk (tumor size ≤2 cm (T1), lymph node-negative (N0), and grade I); intermediate risk (tumor size >2 cm to ≤5 cm (T2), lymph node status N1, or grade II); and high risk (tumor size >5 cm (T3), lymph node status ≥N2, or grade III). Data are presented as number (percentage). Statistical analysis was performed using Pearson’s chi-square test, and a p-value <0.05 was considered statistically significant. χ² = chi-square value; df = degrees of freedom; n = number of cases; T = tumor size category; N = lymph node status

Risk category*	Vimentin positive, *n* (%)	Vimentin negative, *n* (%)	Total, *n* (%)	*χ*²	*P*-value
Low risk	0 (0.0)	15 (100.0)	15 (100.0)	22.84	<0.001
Intermediate risk	6 (15.0)	34 (85.0)	40 (100.0)		
High risk	11 (73.3)	4 (26.7)	15 (100.0)		

When categorized into favorable and unfavorable prognostic groups, vimentin expression was significantly higher in the unfavorable group (Yates χ² = 16.21, *P* < 0.001) (Table 6).

**Table 6:** Association of vimentin expression with overall prognostic category (n = 70). Vimentin expression was assessed by immunohistochemistry and categorized as positive or negative based on cytoplasmic staining. Prognostic categories were defined as follows: favorable (tumor size ≤5 cm (T1/T2), lymph node status N0/N1, and histological grade I/II) and unfavorable (tumor size >5 cm (T3) or lymph node status ≥N2 or histological grade III). Data are expressed as number (percentage). Statistical analysis was performed using the chi-square test with Yates correction for continuity, and a p-value <0.05 was considered statistically significant. χ² = chi-square value; df = degrees of freedom; n = number of cases; T = tumor size category; N = lymph node status

Prognostic category	Vimentin positive, *n* (%)	Vimentin negative, *n* (%)	Total, n (%)	*χ*²	*P*-value
Favorable (T1/T2 + N0/N1 + Grade I/II)	6 (11.3)	47 (88.7)	53 (100.0)	16.21	<0.001
Unfavorable (T3 or ≥N2 or Grade III)	11 (64.7)	6 (35.3)	17 (100.0)		

Predictors of vimentin expression

Logistic regression analysis revealed that histological grade III was the strongest predictor of vimentin expression (odds ratio (OR) = 24.29, *P* < 0.001), followed by lymph node positivity (OR = 3.62, *P* = 0.041), whereas tumor size did not show a statistically significant association (Table 7).

**Table 7:** Logistic regression analysis for predictors of vimentin positivity in invasive breast carcinoma (n = 70). Univariate logistic regression analysis was performed to identify predictors of vimentin positivity. Vimentin expression (positive/negative) was taken as the dependent variable. Independent variables included tumor size, lymph node status, and histological grade. Tumor size was categorized as ≤5 cm versus >5 cm; lymph node status as negative (N0) versus positive (N1-3); and histological grade as grade I/II versus grade III. OR with 95% CI were calculated, and a *P*-value <0.05 was considered statistically significant. OR = odds ratio; CI = confidence interval; n = number of cases; N = lymph node status; T = tumor size category.

Variable	Category	Vimentin positive, *n*/*N* (%)	Odds ratio (OR)	95% CI	*P*-value
Tumor size	≤5 cm	5/31 (16.1)	Reference	-	-
	>5 cm	12/39 (30.8)	2.31	0.71-7.47	0.162
Lymph node status	Negative (N0)	4/32 (12.5)	Reference	-	-
	Positive (N1-N3)	13/38 (34.2)	3.62	1.05-12.48	0.041
Histological grade	Grade I/II	7/57 (12.3)	Reference	-	-
	Grade III	10/13 (76.9)	24.29	5.39-109.4	<0.001

## Discussion

Vimentin is a type III intermediate filament protein that is normally expressed in mesenchymal cells and has emerged as an important marker of epithelial-mesenchymal transition (EMT) in epithelial malignancies [5,20]. In breast carcinoma, aberrant expression of vimentin reflects loss of epithelial differentiation and acquisition of a more migratory, invasive, and biologically aggressive phenotype [6,8]. In the present study, vimentin expression was identified in 17 of 70 cases (24.28%) of invasive breast carcinoma. This frequency is in close agreement with several previous studies. Badowska-Kozakiewicz and Budzik observed positivity in 38 of 179 cases (21.2%), while Cattoretti et al. reported vimentin positivity in approximately 49 of 196 cases (25.0%) [15,21]. Similarly, Hemalatha et al. found vimentin expression in 9 of 50 cases (18.0%), which is almost identical to the present findings [17]. This consistency across studies supports the observation that vimentin is expressed only in a subset of invasive breast carcinomas, but when present, it is often associated with unfavorable pathological features.

In the present study, vimentin expression showed an increasing trend with increasing tumor size. None of the tumors measuring ≤2 cm [0 of 3 cases (0.0%)] showed positivity. In contrast, expression was observed in 5 of 28 cases (17.85%) in tumors measuring >2 to ≤5 cm, and increased further to 12 of 39 cases (30.76%) in tumors measuring >5 cm. Although this association was not statistically significant in the current analysis, the observed trend suggests that vimentin expression may be linked to increasing tumor burden and invasive potential. These findings are comparable to those of Chen et al., who demonstrated that vimentin immunopositivity correlated with larger tumor size in breast cancers of young women [14]. Heatley et al. also reported an association between vimentin expression and adverse pathological parameters, including tumor size [12]. Although an increasing trend of vimentin expression was observed with larger tumor size, this association did not reach strong statistical significance and should therefore be interpreted with caution [4]. However, some authors, including Gogoi et al. and Hemalatha et al.​​​​​​​, did not observe a significant relationship between vimentin expression and tumor size [16,17]. Such differences may be due to variations in sample size, scoring systems, patient population, or tumor subtype composition.

A similar trend was observed with lymph node status in the present study. Vimentin positivity was identified in 4 of 32 node-negative tumors (12.50%), 3 of 17 N1 tumors (17.64%), 8 of 18 N2 tumors (44.44%), and 2 of 3 N3 tumors (66.66%). When grouped broadly, vimentin expression was present in 13 of 38 lymph node-positive tumors (34.21%) compared with 4 of 32 lymph node-negative tumors (12.50%). Although the association did not achieve statistical significance (*P* = 0.06). Although a higher proportion of vimentin positivity was observed in node-positive tumors, the association did not achieve statistical significance and should be interpreted cautiously. This finding is biologically plausible, as vimentin is closely linked to EMT, a process that enhances tumor cell motility, invasion, and dissemination [5,8,20]. Heatley et al. observed an association between vimentin expression and the number of involved lymph nodes [12]. On the other hand, Gogoi et al. and Hemalatha et al. did not find a significant association between vimentin expression and nodal metastasis [16,17]. The lack of statistical significance in the present study may be explained by the relatively small number of cases in higher nodal categories, particularly N3 disease (*n* = 3), which limits statistical power. Nonetheless, the steadily increasing proportion of vimentin-positive tumors with higher nodal burden suggests that this marker may still have practical prognostic relevance.

Although survival data were not available in the present study, the statistically significant association of vimentin expression with composite high-risk histopathological categories (*P* < 0.001) suggests its potential role as a surrogate marker of aggressive tumor biology. However, its direct prognostic value in terms of survival outcomes cannot be established and requires further longitudinal studies. However, these composite categories are intended only for integrative analysis of established parameters and should not be interpreted as independent prognostic models.

The most striking finding of the present study was the strong association between vimentin expression and histological grade. None of the Grade I tumors [0 of 19 cases (0.0%)] showed vimentin positivity, whereas positivity was seen in 7 of 38 Grade II tumors (18.42%) and 10 of 13 Grade III tumors (76.92%). This clear stepwise increase in expression with worsening differentiation strongly indicates that vimentin is associated with poorly differentiated and biologically aggressive breast carcinomas. This observation is in concordance with multiple earlier studies. Raymond and Leong reported that vimentin was preferentially expressed in high-grade infiltrating ductal carcinomas [10]. Domagala et al. similarly found that vimentin was preferentially expressed in high-grade ductal breast carcinomas and in tumors with adverse biological features [11]. Chen et al. demonstrated that vimentin was significantly associated with higher histological grade, and Badowska-Kozakiewicz and Budzik also found that vimentin-positive tumors were more often high-grade cancers [14,15]. Hemalatha et al. likewise concluded that vimentin-positive tumors were associated with high-grade malignancy and increased tumor proliferation [17]. Thus, the present findings strongly reinforce the concept that vimentin is a marker of dedifferentiation and tumor aggressiveness in breast carcinoma.

The findings of the present study should be interpreted cautiously. Although statistically significant associations were observed, particularly with histological grade and composite risk categories, the absence of survival and follow-up data limits the ability to establish direct prognostic implications. Therefore, vimentin expression should be considered a marker associated with adverse pathological characteristics rather than a definitive prognostic indicator.

The biological explanation for these findings likely lies in the central role of vimentin in epithelial-mesenchymal transition. EMT is increasingly recognized as a crucial mechanism in tumor invasion and metastasis, during which epithelial cells lose polarity and intercellular adhesion and acquire mesenchymal features that facilitate migration and dissemination [5,20]. Vimentin is a hallmark protein of EMT. It contributes not only as a marker but also as an active regulator of cytoskeletal remodeling, cellular motility, and focal adhesion dynamics [6,8]. Experimental studies have shown that vimentin promotes breast cancer cell migration, invasion, and mechanical adaptability, thereby facilitating metastatic behavior [6,22]. Therefore, the association of vimentin with larger tumor size, increasing nodal burden, and higher histological grade observed in the present study is consistent with its established biological role in tumor progression.

It is important to note that the present study design is observational and cross-sectional in nature, and therefore supports only correlation rather than causation. While significant associations were identified, particularly with histological grade, these findings do not imply a direct causal role of vimentin in tumor progression.

From a clinical standpoint, the findings of this study suggest that vimentin may serve as a potential adjunct marker associated with aggressive histopathological features. Histological grade, tumor size, and nodal status remain the backbone of pathological prognostication in breast cancer [3,4], and the addition of a biologically meaningful immunohistochemical marker such as vimentin may provide further insight into tumor aggressiveness. Although vimentin is not currently part of routine prognostic panels in standard breast pathology practice, its association with poor pathological parameters indicates potential value in identifying tumors with a more aggressive phenotype.

The present study has several limitations. It is a single-center study with a relatively small sample size, which may limit the generalizability of the findings. Higher nodal categories, particularly N3 disease, were underrepresented, which may have affected the statistical power for subgroup analyses. The absence of molecular subtype correlation, including hormone receptor and HER2 status, limits the biological interpretation of vimentin expression. Furthermore, the lack of follow-up and survival data precludes direct assessment of prognostic significance. Although composite risk stratification was utilized to integrate established histopathological parameters, these categories should be considered exploratory and interpreted with caution, as they do not replace validated prognostic models. While efforts were made to standardize immunohistochemical evaluation, including defined scoring criteria and interobserver assessment, variability in staining interpretation cannot be entirely excluded.

## Conclusions

Vimentin expression in invasive breast carcinoma is associated with adverse histopathological features, particularly higher histological grade and increased nodal burden. It may serve as an adjunct marker reflecting aggressive tumor biology; however, further studies incorporating survival analysis are required to establish its definitive prognostic value.
